# O-Glycosylating Enzyme GALNT2 Predicts Worse Prognosis in Cervical Cancer

**DOI:** 10.3389/pore.2022.1610554

**Published:** 2022-08-30

**Authors:** Lixia Zhou, Huiqin Wu, Xingli Bai, Shuyun Min, Jiawen Zhang, Cunli Li

**Affiliations:** ^1^ Department of Obstetrics and Gynecology, Jiading Branch of Shanghai General Hospital, Shanghai Jiao Tong University School of Medicine, Shanghai, China; ^2^ Department of Obstetrics and Gynecology, Shanghai Songjiang District Maternal and Child Health Hospital, Shanghai, China; ^3^ Department of Obstetrics and Gynecology, Shanghai General Hospital, Shanghai Jiao Tong University School of Medicine, Shanghai, China; ^4^ Reproductive Medicine Center, Department of Obstetrics and Gynecology, Shanghai General Hospital, Shanghai Jiao Tong University School of Medicine, Shanghai, China

**Keywords:** survival, cervical cancer, GALNT2, anti-tumor immune response, immune-related biomarker

## Abstract

Identification of novel biomarkers is helpful for the diagnosis and treatment of cervical cancer. Mucin glycosylating enzyme GALNT2 modulates mucin O-glycosylation, and has been revealed as a regulator of tumorigenesis in various cancers. However, the expression pattern of GALNT2 in cervical cancer is still unclear. In this study, we demonstrated that the mRNA expression and protein level of GALNT2 were increased in cervical high-grade intraepithelial neoplasia and tumor tissues compared with normal cervix tissues. Kaplan-Meier plotter showed that overexpression of GALNT2 was associated with worse overall survival in TCGA cohort (*p* < 0.001, HR = 2.65, 95% CI = 1.62–4.34) and poor disease free survival in GSE44001 cohort (*p* = 0.0218, HR = 2.15, 95% CI = 1.14–4.06). In addition, GSEA analysis showed that various immune-related pathways were closely related to the expression of GALNT2 in cervical cancer. Moreover, co-expression of GALNT2 and IL1A, IL1B, IL11, CXCL1, CXCL2, CXCL5, CXCL6, CXCR1, or CCR3 predicted poor overall survival, and the expression of GALNT2 also affected the prognostic value of CD47, CD274, CD276, CSF1R, TNFSF9, and TNFSF11 in cervical cancer patients. These findings suggest that GALNT2 might be used as a prognostic biomarker in cervical cancer.

## Introduction

Cervical cancer is one of the most serious health threats in women worldwide [1]. Although great progress has been made in the diagnosis and treatment of cervical cancer in the past few decades, the prognosis of patients with cervical cancer is still unsatisfactory due to the late detection, recurrence and metastasis [2]. Recently, immunotherapy has received extensive attention in the treatment of cervical cancer. However, the efficacy of antitumor immunotherapy in recurrent and advanced cervical cancer remains to be improved [3]. Therefore, it is urgent to find out key immune-related biomarkers of cervical cancer and reveal their potential mechanisms in the occurrence and development of cervical cancer.

Protein glycosylation is one of the most common post-translational modifications. There are eight glycosylation pathways in mammals, among which N-glycosylation and O-glycosylation are the most well-known modifications [4]. Abnormal glycosylation affects a variety of cellular characteristics, such as cell differentiation, proliferation, apoptosis, invasion, migration and immune responses, and has been found as a promising target for cancer therapeutics [4, 5]. Previous studies revealed that glycosylation pattern could play roles in diagnosis and prognosis of cervical disease [6, 7]. Solórzano et al. [8] reported a complex of overexpressed glycosylated proteins, which plays relevant roles in the invasive capacity of cervical cancer, by using Machaerocereus eruca (MeA). The events of glycosylation are mainly mediated by glycosyltransferases and glycosidases, and the role of glycosyltransferase in cancers has been paid more and more attention. For instance, overexpression of glycosyltransferase ST6Gal-I in epithelial cell is correlated with premalignant progression [9], and activates epithelial to mesenchymal transition (EMT) pathways [10]. Polypeptide N-Acetylgalactosaminyltransferase 14 (GALNT14) has been identified as a putative driver of cancer metastasis, and predicts poor patient survival [11, 12]. However, the expression pattern of glycosyltransferases in cervical cancer is still largely unknown.

Here, we focus on the expression pattern of mucin glycosylating enzyme GALNT2 in cervical cancer. Public databases and our immunohistochemistry staining demonstrated that GALNT2 expression was increased in cervical cancer compared with normal cervix tissues. Moreover, high expression of GALNT2 was not only associated with poor overall survival and disease free survival, but also affected the prognostic value of specific cytokines, chemokines and immune modulatory molecules. Our analysis suggested that GALNT2 might be a potential unfavorable factor in cervical cancer.

## Materials and Methods

### Human Tissue Specimens and Immunohistochemical Analysis

The human tissue specimen microarrays including 10 normal cervix, 27 low-grade squamous intraepithelial lesions (LSIL), 55 high-grade squamous intraepithelial lesions (HSIL), 31 cervical squamous cell carcinoma (SCC) and paired adjacent normal tissues were purchased from Shanghai Zuocheng Biotechnology (Shanghai, China). The pathological diagnoses were done in accordance with the International Federation of Gynecology and Obstetrics classification system. This study was approved by the Institutional Ethics Committee of Shanghai General Hospital (reference 2021SQ263).

The IHC procedure was performed to determine the protein level of GALNT2. Briefly, paraffin-embedded tissue microarrays were deparaffinized and rehydrated. Then the microarrays were placed in sodium citrate buffer (pH 6.0) and boiled for 5 min in a pressure cooker, following treatment with H_2_O_2_ at room temperature for 10 min. After washing with phosphate-buffered saline (PBS), the microarrays were incubated with GALNT2 rabbit polyclonal antibody (diluted to 1:100; Abcam, Cambridge, United Kingdom) at 4°C overnight. The next day, these microarrays were incubated with a secondary antibody (goat anti-rabbit IgG H&L, Abcam, Cambridge, United Kingdom) conjugated to a polymer coated with horseradish peroxidase (HRP) at room temperature for 60 min. The IHC staining of the microarrays was examined by two separate experienced pathologists (Jiangtao Xu and Yinze Wei) using a microscope (Olympus, Tokyo, Japan) in five randomly selected representative fields.

The IHC score was determined by multiplying the staining extent by the staining intensity. The staining extent was scored as <10% (0), 10%–25% (1), 25%–50% (2), 50%–75% (3), and >75% (4). The staining intensity was divided into four score ranks: no staining (0), light brown (1), brown (2), and dark brown (3).

### Bioinformatic Analysis

The Gene Expression Omnibus (GEO) was used to evaluate the gene expression in different tissues. GSE9750 was used to analyze the mRNA expression of 101 glycosyltransferases between 33 cervical cancer and 24 normal cervical epithelium tissues. GSE7803 was used to analyze the mRNA expression of GALNT2 between 21 invasive squamous cell carcinomas and 10 normal squamous cervical epithelium samples. GSE6791 was used to analyze the mRNA expression of GALNT2 between 20 cervical cancer and 8 normal cervical samples. GSE7410 was used to analyze the mRNA expression of GALNT2 between 40 cervical cancer and 5 normal cervical samples. GSE44001 was used to analyze disease free survival in 300 samples of early cervical cancer.

UALCAN web tool (http://ualcan.path.uab.edu/) was used to assess the expression of GALNT2 in cervical cancer [13].

Survival analyses were performed by using Kaplan-Meier estimator from the Kaplan-Meier plotter (http://kmplot.com/analysis/) based on The Cancer Genome Atlas (TCGA) repository [14].

Gene set enrichment analysis (GSEA) of KEGG pathway, Panther pathway, Gene Ontology (GO) biological process and GO molecular function was performed by the LinkedOmics platform (http://www.linkedomics.org) based on TCGA repository [15].

HALLMARK pathway in transcriptome levels between high and low GALNT2 expression in cervical cancer was evaluated *via* GSEA v3.0 software [16, 17]. The number of permutations was set at 100. Enrichment results satisfying a nominal P-value <0.05 was considered statistically significant.

TIMER2.0 web tool (http://timer.cistrome.org/) was used to analyze the differential expression between tumor and adjacent normal tissues for GALNT2 across different cancer types [18].

Single-sample gene set enrichment analysis (ssGSEA) algorithm was conducted to evaluate the infiltration of immune cells by the R package GSVA.

### Statistical Analysis

The normal distribution was tested by D'Agostino-Pearson or Kolgomorov-Smirnov’s test, and the comparison of gene expression level between different groups was performed by using Student’s *t* tests or Mann-Whitney test depending on the nature of distribution. ANOVA or non-parametric ANOVA analysis was used in multiple comparisons depending on the nature of distribution. Spearman correlation analysis was performed between the expression of GALNT2 and different genes. The correlation coefficients of the results were shown. Survival rate was analyzed by using the Kaplan-Meier method with log-rank test. Hazard ratio (HR) and 95% confidence interval (CI) were calculated to evaluate the survival difference. Univariate and multivariate Cox proportional hazard regression analyses were performed to confirm independent factors for predicting the prognosis of cervical cancer. All statistical analyses and graphs were performed by using GraphPad Prism (Version 9.0, GraphPad Software Inc., United States). *p* < 0.05 was considered statistically significant.

## Results

### The Expression of GALNT2 is Increased in Cervical Cancer

**FIGURE 1:**
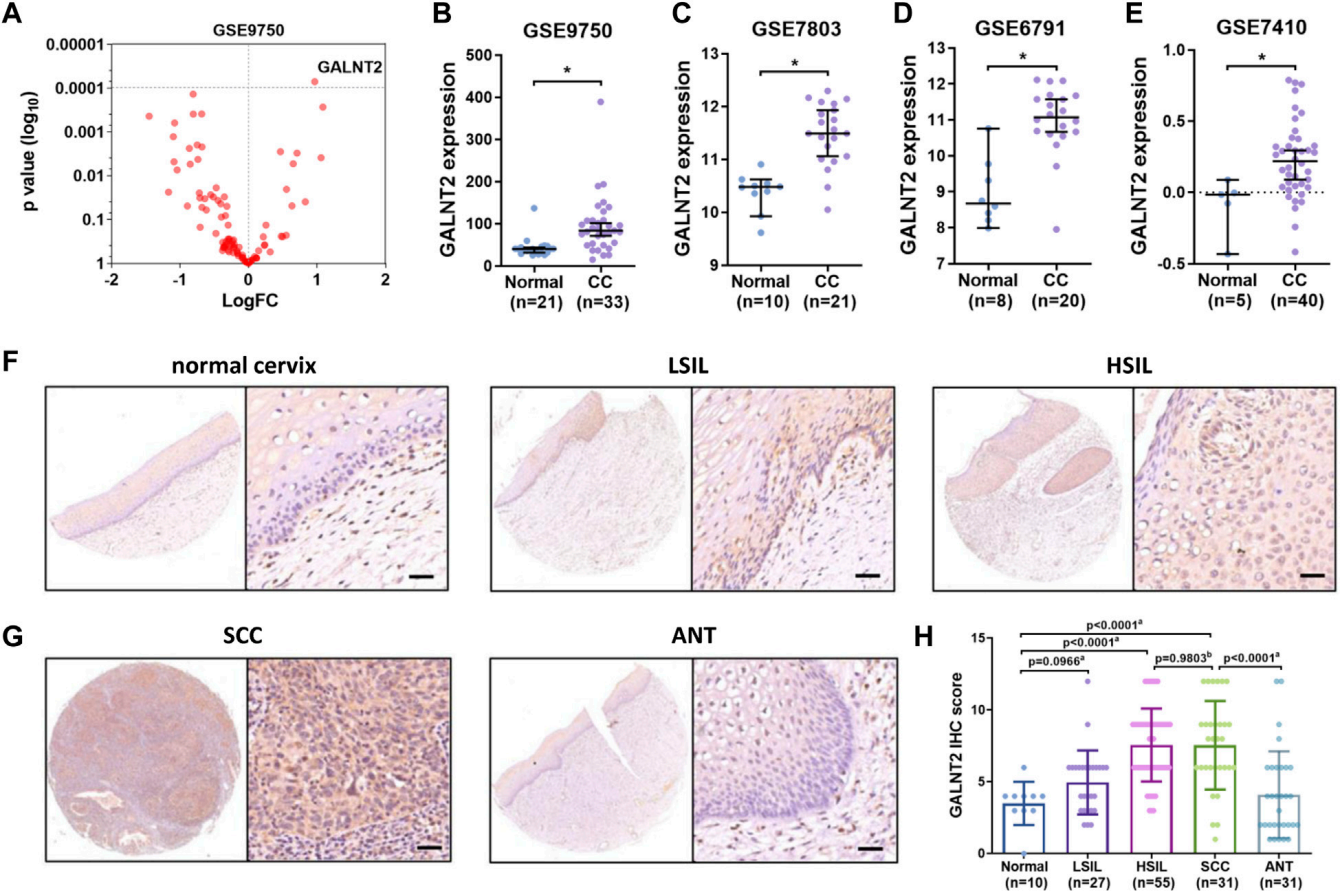
The expression of GALNT2 is increased in cervical cancer. **(A)** The expression of 101 different glycosyltransferases in cervical cancer from GSE9750, the p-value and logFC were shown. **(B)** The expression of GALNT2 between 33 cervical cancer and 24 normal cervical epithelium tissues from GSE9750 (D'Agostino-Pearson test, K2 = 43.41, *p* < 0.0001; Mann-Whitney test, *p* < 0.0001). **(C)** The expression of GALNT2 between 21 invasive squamous cell carcinomas and 10 normal squamous cervical epithelium samples from GSE7803 (D'Agostino-Pearson test, K2 = 4.026, *p* = 0.1336; t test, *t* = 5.355, *p* < 0.0001). **(D)** The expression of GALNT2 between 20 cervical cancer and 8 normal cervical samples from GSE6791 (D'Agostino-Pearson test, K2 = 2.5, *p* = 0.2865; t test, *t* = 5.076, *p* < 0.0001). **(E)** The expression of GALNT2 between 40 cervical cancer and 5 normal cervical samples from GSE7410 (Kolgomorov-Smirnov’s test, *p* > 0.1; t test, *t* = 2.503, *p* = 0.0162). **(F)** The protein level of GALNT2 in normal cervix and squamous intraepithelial lesion tissues by using IHC analysis. Scale bar = 50 μm. **(G)** The protein level of GALNT2 in cervical cancer and adjacent non-tumor (ANT) tissues by using IHC analysis. Scale bar = 50 μm. **(H)** The IHC score of GALNT2 in normal cervix, squamous intraepithelial lesion and cervical cancer tissues (D'Agostino-Pearson test, *p* > 0.1; ^a^ Mann-Whitney test, ^b^ t test, non-parametric ANOVA). CC, cervical carcinoma; LSIL, low-grade squamous intraepithelial lesion; HSIL, high-grade squamous intraepithelial lesion; SCC, squamous cell carcinoma; ANT, adjacent normal tissue; IHC, immunohistochemistry.

In order to explore the expression pattern of different glycosyltransferases in cervical cancer, we first analyzed the expression alterations of 101 glycosyltransferases between normal cervix and cervical cancer tissues by using GEO database. Compared with normal cervix tissues, the expressions of several glycosyltransferases in cervical cancer tissues were upregulated, including GALNT2, POGLUT1, EXT1, B3GAT3, B4GALNT1 and UGT8 (GSE9750, Figures 1A,B). As GALNT2 is the most altered glycosyltransferase, we chose GALNT2 as the target gene and tried to explore the role of GALNT2 in cervical cancer.

Then we analyzed other GEO databases, and showed that GALNT2 was increased in cervical cancer samples (GSE7803, GSE6791, GSE7410, Figures 1C–E). Moreover, UALCAN showed that the expression of GALNT2 in stage 1-4 was higher than that in normal samples, whereas there was no difference among different stages (Supplementary Figure S1A). Based on tumor histology, the expression of GALNT2 in squamous cell cancer was higher than that in normal samples, whereas there was no difference between normal and adenosquamous carcinoma, mucinous adenocarcinoma or endometrioid adenocarcinoma (Supplementary Figure S1B). Moreover, GALNT2 expression was higher in chemotherapy partial response group and progressive disease group than that in complete response group based on TCGA-CESC data (Supplementary Figure S1C).

Next, we confirmed these results by using IHC. As shown in Figures 1F–H, the GALNT2 protein was localized in both cytoplasm and nucleus. Compared with normal cervix, the positive staining pattern of GALNT2 was increased according to the grades of the intraepithelial lesions. The protein level of GALNT2 in HSIL was higher than that in normal cervix and LSIL, whereas there was no difference between normal cervix and LSIL (Figures 1F–H). In the cases of SCC, the staining signal of GALNT2 was mainly positive or strong positive, and the IHC score of GALNT2 was significantly higher than adjacent normal tissues (Figures 1G,H). However, there was no difference between HSIL and SCC tissues (Figure 1H). In addition, the expression of GALNT2 was also upregulated in various cancer types, such as breast invasive carcinoma (BRCA), colon adenocarcinoma (COAD), head-neck squamous cell carcinoma (HNSC), kidney renal clear cell carcinoma (KIRC), lung adenocarcinoma (LUAD), lung squamous cell carcinoma (LUSC) and stomach adenocarcinoma (STAD) (Supplementary Figure S1D).

### High Expression of GALNT2 Predicts Poor Prognosis in Cervical Cancer

**FIGURE 2:**
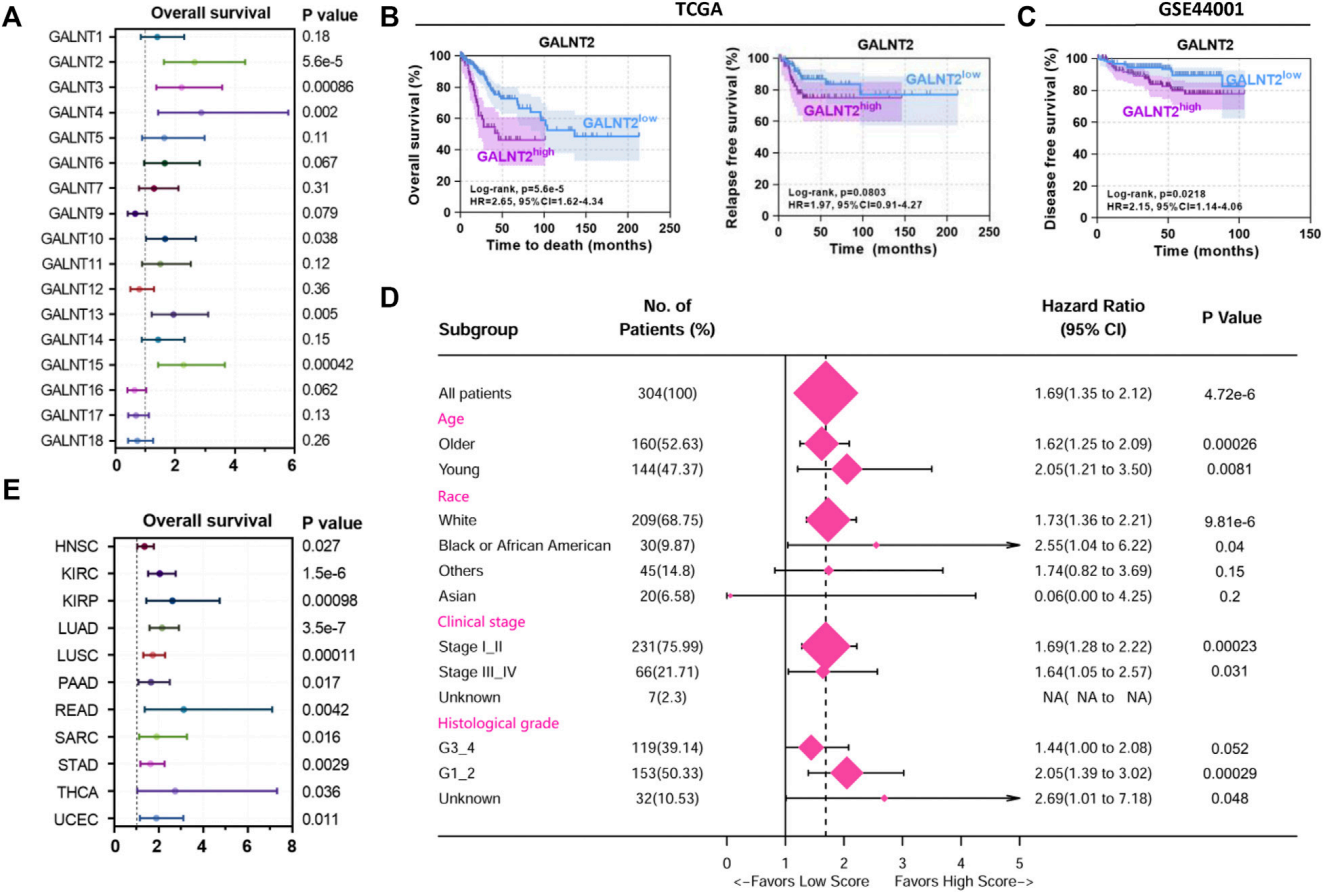
High expression of GALNT2 predicts poor prognosis in cervical cancer. **(A)** The distribution of hazard ratios across GalNAc-T subfamily members in patients with cervical cancer from Kaplan-Meier plotter (http://kmplot.com/analysis/). **(B)** Survival analysis of GALNT2 on overall survival (OS) or relapse-free survival (RFS) of cervical cancer patients from TCGA data *via* Kaplan-Meier plotter. **(C)** Survival analysis of GALNT2 on disease free survival (DFS) of cervical cancer patients from GSE44001. **(D)** Univariate Cox hazard ratio analysis showed that the expression of GALNT2 was statistically different in the subgroups classified by age, race, clinical stage and histological grade based on TCGA data. **(E)** The distribution of hazard ratios of GALNT2 in patients with different cancer types from Kaplan-Meier plotter. HNSC, head and neck squamous cell carcinoma; KIRC, kidney renal clear cell carcinoma; KIRP, kidney renal papillary cell carcinoma; LUAD, lung adenocarcinoma; LUSC, lung squamous cell carcinoma; PAAD, pancreatic adenocarcinoma; READ, rectum adenocarcinoma; SARC, sarcoma; STAD, stomach adenocarcinoma; THCA, thyroid carcinoma; UCEC, uterine corpus endometrial carcinoma.

Then we evaluated the prognostic value of GalNAc-T subfamily members based on TCGA cervical cancer cohort. Kaplan-Meier analysis showed that high expression levels of GALNT2, GALNT3, GALNT4, GALNT10, GALNT13 and GALNT15 were associated with worse overall survival (Figure 2A), among which GALNT2 was also the most significant prognostic indicator (*p* = 5.6e-5, HR = 2.65, 95% CI = 1.62–4.34, Figure 2B). Similarly, high expression of GALNT2 was associated with poor relapse-free survival (RFS), although the difference was not statistically significant (*p* = 0.08, HR = 1.97, 95% CI = 0.91–4.27, Figure 2B). In GSE44001 cohort (*n* = 300), high expression of GALNT2 was correlated with poor disease free survival (DFS) (*p* = 0.0218, HR = 2.15, 95% CI = 1.14–4.06, Figure 2C). Moreover, the association of GALNT2 expression with OS in different clinical features (age, race, clinical stage and histological grade) was examined by univariate Cox analysis, and the results showed that increased expression of GALNT2 indicated worse survival (Figure 2D). In addition, pan-cancer analysis showed that the increased expression of GALNT2 also predicted unfavorable prognosis in several other cancers, including HNSC, KIRC, LUAD, LUSC, STAD, rectum adenocarcinoma (READ) and uterine corpus endometrial carcinoma (UCEC) (Figure 2E).

### GSEA and Correlation Analysis of Immune Infiltration of GALNT2 in Cervical Cancer

**FIGURE 3:**
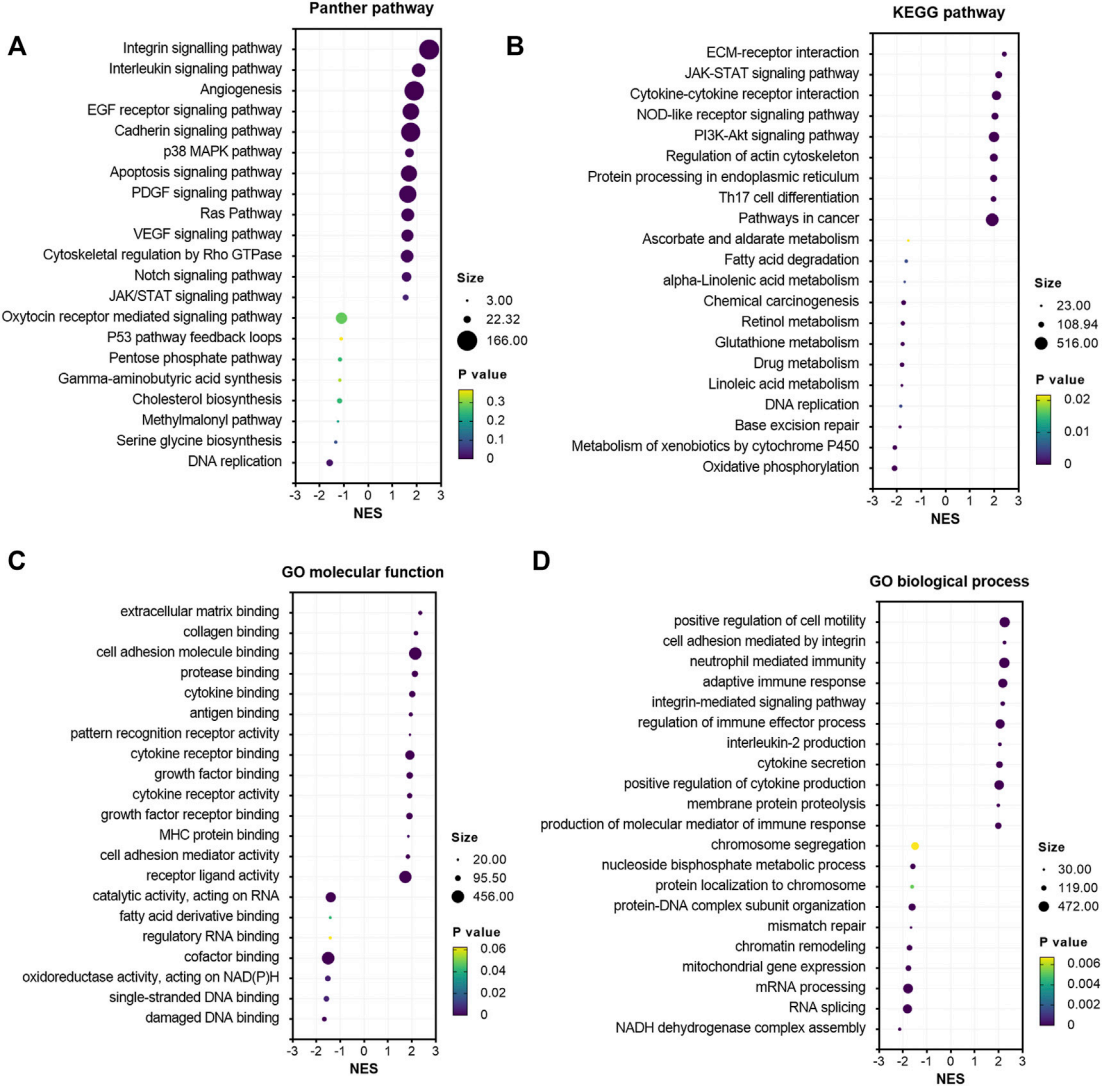
Gene set enrichment analysis of GALNT2 in cervical cancer. **(A)** The correlation of Panther pathways and GALNT2 analyzed by GSEA *via* LinkedOmics. **(B)** The correlation of KEGG pathways and GALNT2 analyzed by GSEA via LinkedOmics. **(C)** The correlation of GO molecular function and GALNT2 analyzed by GSEA *via* LinkedOmics. **(D)** The correlation of GO biological process and GALNT2 analyzed by GSEA *via* LinkedOmics.

To explore the potential oncogenic pathways by which GALNT2 are involved in cervical cancer, we used GSEA to analyze the correlation between GALNT2 expression and oncogenic pathways. Panther pathway analysis demonstrated that the expression of GALNT2 in cervical cancer was correlated with several tumorigenesis-related pathways, such as integrin signaling pathway, interleukin signaling pathway, EGF receptor signaling pathway, cadherin signaling pathway, Ras pathway and Notch signaling pathway (Figure 3A). Similarly, KEGG pathway enrichment showed that ECM receptor interaction, JAK-STAT signaling pathway, cytokine-cytokine receptor interaction, NOD-like receptor signaling pathway, PI3K-Akt signaling pathway and Th17 cell differentiation were associated with GALNT2 expression (Figure 3B). Moreover, HALLMARK oncogenic pathway analysis demonstrated that the expression of GALNT2 was correlated with apical junction, angiogenesis, PI3K/Akt mTOR signaling, KRAS signaling, IL6/JAK/STAT3 signaling and inflammatory response (Supplementary Figure S2). Additionally, we also performed Gene Ontology (GO) enrichment analysis. There also existed statistically significant differences in terms of the enrichment score of immune-related gene sets correlated with cytokine binding, cytokine receptor binding, cytokine receptor activity, antigen binding, adaptive immune response, regulation of immune effector process, cytokine secretions and production of molecular mediator of immune response (Figures 3C,D). Furthermore, correlation analysis demonstrated that GALNT2 expression was associated with natural killer cell and regulatory T cell infiltration in both TCGA and GSE44001 cohorts, whereas other correlations were not consistent in these cohorts (Supplementary Figure S3).

### Correlation Analysis of GALNT2 Expression With Cytokines and Chemokines

**FIGURE 4:**
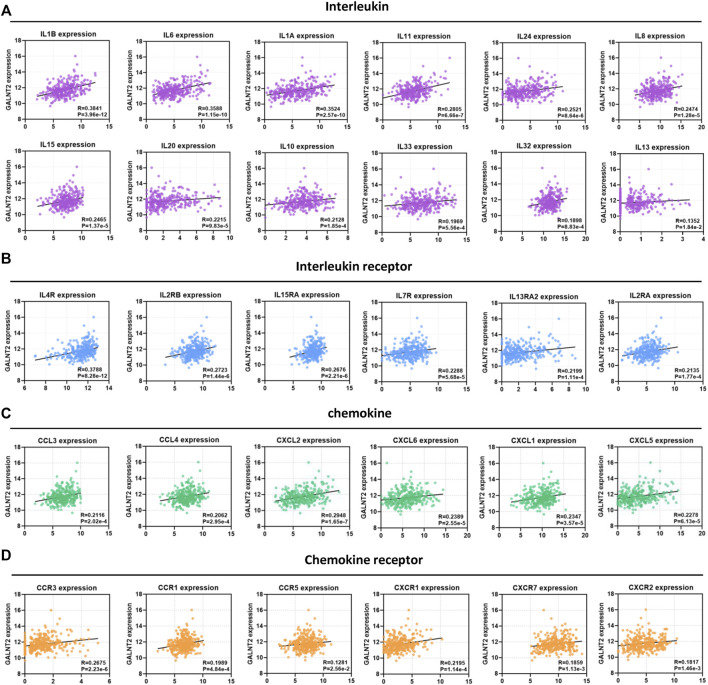
Correlation analysis of GALNT2 expression with cytokines and chemokines. **(A)** Correlation of GALNT2 expression with interleukins (IL1B, IL6, IL1A, IL11, IL24, IL8, IL15, IL20, IL10, IL33, IL32, and IL13). **(B)** Correlation of GALNT2 expression with interleukin receptors (IL4R, IL2RB, IL15RA, IL7R, IL13RA2, and IL2RA). **(C)** Correlation of GALNT2 expression with chemokines (CCL3, CCL4, CXCL2, CXCL6, CXCL1, and CXCL5). **(D)** Correlation of GALNT2 expression with chemokine receptors (CCR3, CCR1, CCR5, CXCR1, CXCR7, and CXCR2).

In view of the important role of cytokines and chemokines in regulating antitumor immunity (Supplementary Table S1), we analyzed the association between GALNT2 expression and cytokines/chemokines. We observed a significant correlation of GALNT2 expression with interleukins, such as IL1B (R = 0.3841, *p* = 3.96e-12), IL6 (R = 0.3588, *p* = 1.15e-10), IL1A (R = 0.3524, *p* = 2.57e-10), IL11 (R = 0.2805, *p* = 6.66e-7), IL24 (R = 0.2521, *p* = 8.64e-6) and IL8 (R = 0.2474, *p* = 1.28e-5) (Figure 4A). The expression of GALNT2 showed significant correlation with the expression of interleukin receptors, such as IL4R (R = 0.3788, *p* = 8.28e-12), IL2RB (R = 0.2723, *p* = 1.44e-6), IL15RA (R = 0.2676, *p* = 2.21e-6) and IL7R (R = 0.2288, *p* = 5.68e-5) (Figure 4B), Moreover, we also found that the expressions of several chemokines, such as CCL3 (R = 0.2116, *p* = 2.02e-4), CCL4 (R = 0.2062, *p* = 2.95e-4), CXCL2 (R = 0.2948, *p* = 1.65e-7) and CXCL6 (R = 0.2389, *p* = 2.55e-5), and chemokine receptors, such as CCR3 (R = 0.2675, *p* = 2.23e-6), CCR1 (R = 0.1989, *p* = 4.84e-4), CXCR1 (R = 0.2195, *p* = 1.14e-4) and CXCR7 (R = 0.1859, *p* = 1.13e-3), had a strong association with GALNT2 expression (Figures 4C,D).

### Prognostic Significance of Co-Expression of GALNT2 and Cytokines or Chemokines

**FIGURE 5:**
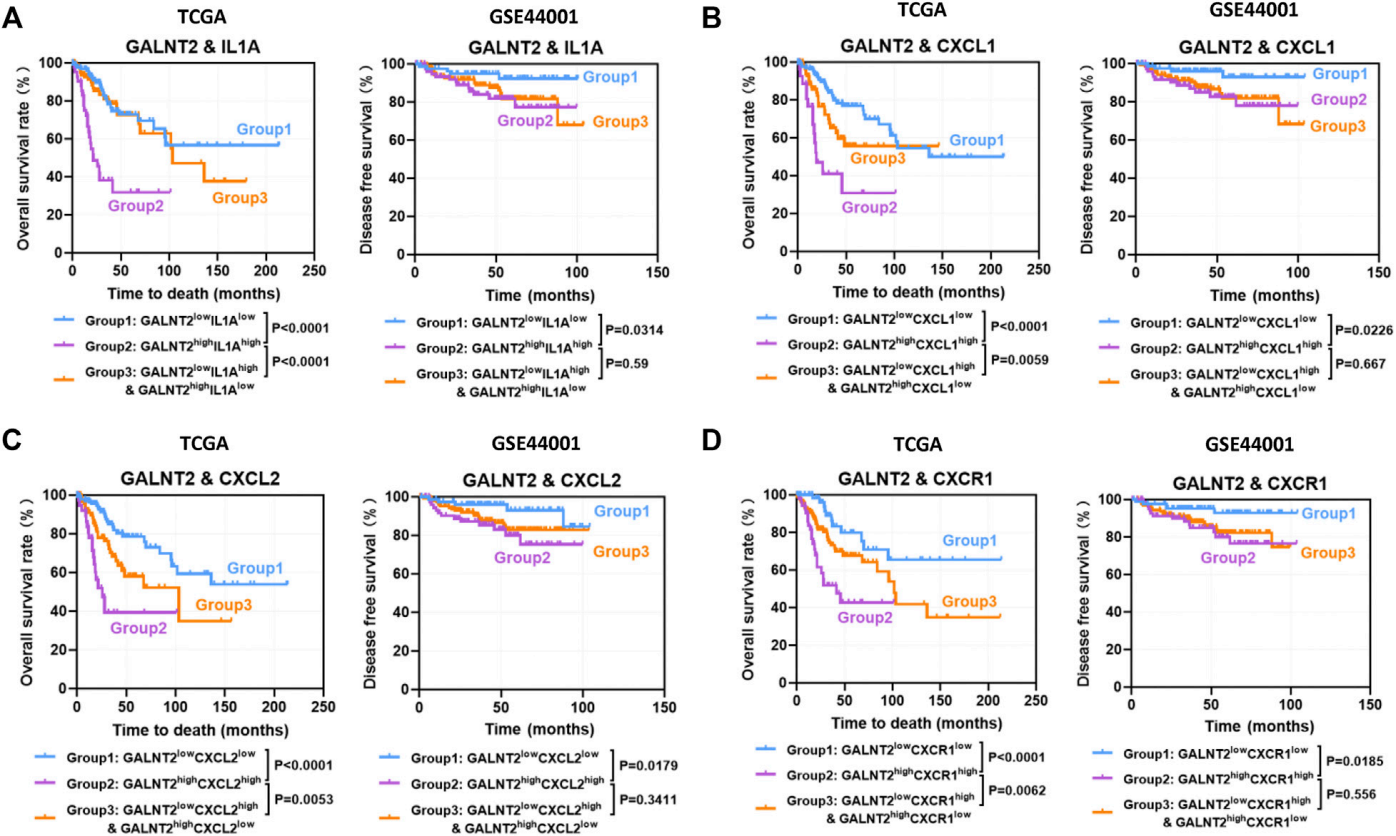
Prognostic significance of co-expression of GALNT2 and cytokines or chemokines. Kaplan-Meier curves were plotted based on the different groups of GALNT2/IL1A **(A)**, GALNT2/CXCL1 **(B)**, GALNT2/CXCL2 **(C)**, and GALNT2/CXCR1 **(D)** in TCGA cohort and GSE44001 cohort.

Then, we evaluated the effect of co-expression of GALNT2 with cytokines or chemokines mentioned above on the prognosis of patients in TCGA cervical cancer cohort. In TCGA cohort, the concomitant high expressions of GALNT2/IL1A was associated with poor overall survival, compared with GALNT2^low^IL1A^low^ group (*p* < 0.0001), GALNT2^low^IL1A^high^ and GALNT2^high^IL1A^low^ group (*p* < 0.0001) (Figure 5A). Similarly, GALNT2^high^IL1B^high^, GALNT2^high^IL11^high^, GALNT2^high^CXCL1^high^, GALNT2^high^CXCL2^high^, GALNT2^high^CXCL5^high^, GALNT2^high^CXCL6^high^, GALNT2^high^CXCR1^high^ or GALNT2^high^CCR3^high^ group predicted worse prognosis of cervical cancer patients, compared with corresponding low expression group or mixed expression group (Figures 5B–D, Supplementary Figure S4). Moreover, high co-expression of GALNT2 and IL1A, CXCL1, CXCL2 or CXCR1 was also associated with worse disease free survival compared with corresponding low expression group in GSE44001 cohort (*p* < 0.05) (Figures 5A–D).

### The Impact of GALNT2 on the Prognostic Assessment of Immune Modulatory Molecules

**FIGURE 6:**
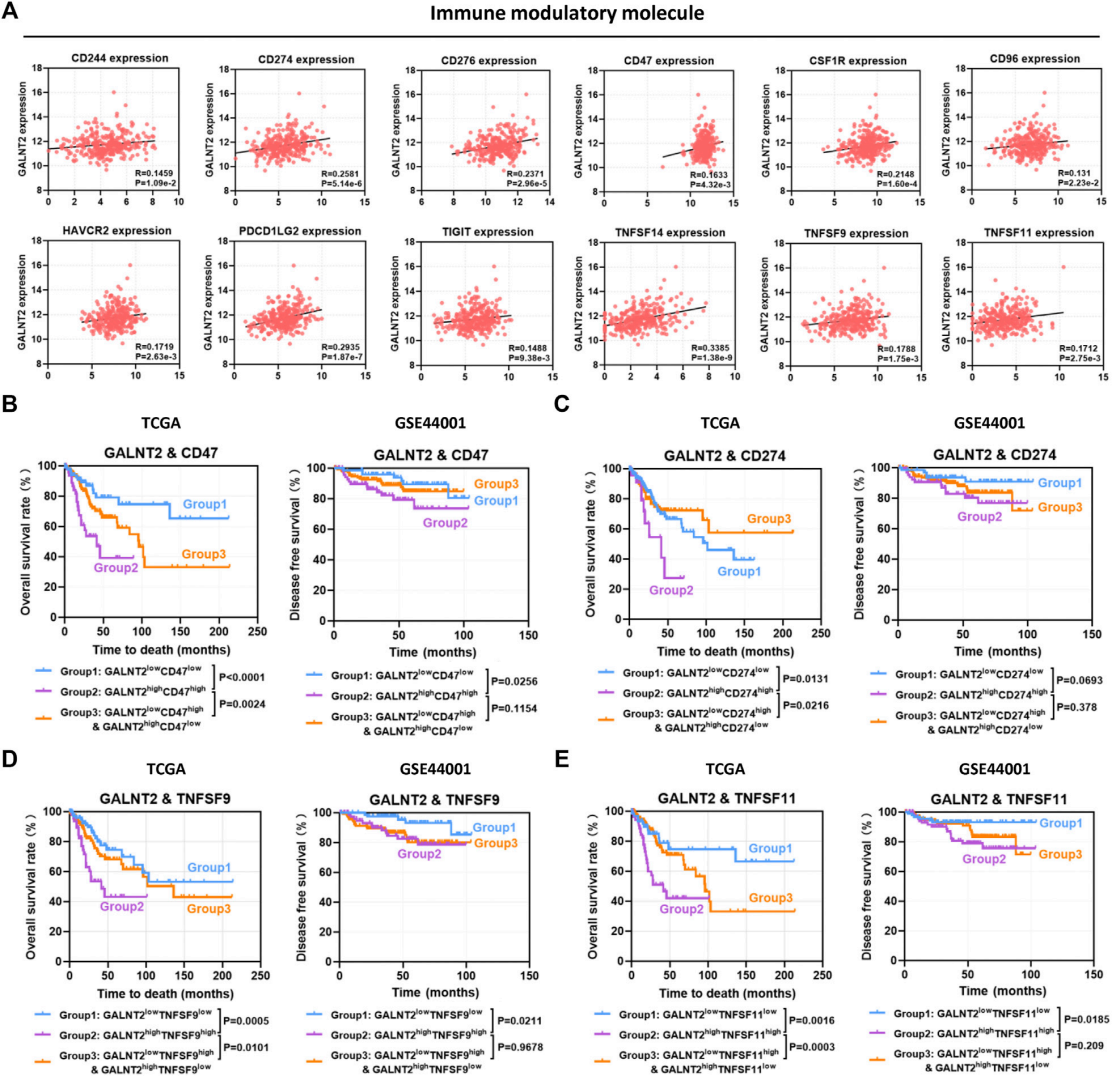
The impact of GALNT2 on the prognostic assessment of immune modulatory molecules. **(A)** Correlation of GALNT2 expression with immune modulatory molecules (CD244, CD274, CD276, CD47, CSF1R, CD96, HAVCR2, PDCD1LG2, TIGIT, TNFSF14, TNFSF9, and TNFSF11). Kaplan-Meier curves were plotted based on the different groups of GALNT2/CD47 **(B)**, GALNT2/CD274 **(C)**, GALNT2/TNFSF9 **(D)** and GALNT2/TNFSF11 **(E)** in TCGA cohort.

Previous studies have confirmed that aberrant expression of immune modulatory molecules affect the antitumor immune response in cervical cancer (Supplementary Table S1), whereas the expressions of several molecules were not related to patients’ prognosis in TCGA cervical cancer cohort (Supplementary Figure S5). To further investigate the impact of GALNT2 on the prognostic assessment of immune modulatory molecules, the co-expression relationship between GALNT2 and immune modulatory molecules were performed by Spearman correlation analysis. As shown in Figure 6A, there was a positive correlation between GALNT2 and CD274 (R = 0.2581, *p* = 5.14e-6), CD276 (R = 0.2371, *p* = 2.96e-5), CD47 (R = 0.1633, *p* = 4.32e-3), CSF1R (R = 0.2148, *p* = 1.60e-4), TNFSF14 (R = 0.3385, *p* = 1.38e-9) or TNFSF9 (R = 0.1788, *p* = 1.75e-3). Kaplan-Meier analysis showed that GALNT2^high^CD47^high^ group was associated with worse prognosis of cervical cancer patients, compared with GALNT2^low^CD47^low^ group (*p* < 0.0001), GALNT2^low^CD47^high^ and GALNT2^high^CD47^low^ group (*p* = 0.0024) (Figure 6B). Similarly, GALNT2^high^CD274^high^, GALNT2^high^TNFSF9^high^ or GALNT2^high^TNFSF11^high^ group predicted poor overall survival of cervical cancer patients compared with corresponding low expression group or mixed expression group, whereas GALNT2^high^CD276^high^ group was associated with worse prognosis compared with GALNT2^low^CD276^low^ group, and GALNT2^high^CSF1R^high^ group was associated with worse prognosis compared with GALNT2^high^CSF1R^low^ and GALNT2^low^CSF1R^high^ group in TCGA cohort (Figures 6C–E, Supplementary Figure S6). Moreover, high co-expression of GALNT2 and CD47, TNFSF9 or TNFSF11 was correlated with poor disease free survival compared with corresponding low expression group in GSE44001 cohort (*p* < 0.05) (Figures 6B,D,E). Additionally, univariate and multivariate Cox proportional hazard regression analyses confirmed that GALNT2 expression and clinical stage were independent factors for predicting the prognosis of cervical cancer, whereas other factors mentioned above had no predictive value in multivariate Cox survival analysis (Supplementary Table S2).

## Discussion

In current study, we showed the expression pattern and prognostic value of O-glycosylating enzyme GALNT2 in cervical cancer. The mRNA expression and protein level of GALNT2 was upregulated in cervical high-grade intraepithelial neoplasia and cervical cancer tissues. Upregulated GALNT2 was associated with shorter survival, and concomitant high expressions of GALNT2 and specific cytokines or chemokines could also predict poor patients’ survival. Importantly, GALNT2 affected the prognostic assessment of immune modulatory molecules in cervical cancer patients.

Glycosyltransferases are responsible for glycan initiation and elongation by catalyzing the formation of glycosidic bond using sugar donors [18]. Sufficient evidences suggest that abnormal expression of glycosyltransferases can alter the formation of glycans, which plays important roles in tumorigenesis [4]. GALNT2 is a member of the glycosyltransferase 2 protein family, which initiates mucin-type O-glycoslation of peptides in the Golgi apparatus [19]. Previous studies have revealed that GALNT2 could influence triglyceride levels, and was involved in type 2 diabetes, hypertension, as well as cancers [20–22]. The dysregulation of GALNT2 contributes to the malignant behavior of hepatocellular carcinoma cells, and enhances the motility of oral squamous cell carcinoma [23, 24], whereas GALNT2 has also been reported to suppress malignant phenotypes in neuroblastoma and gastric adenocarcinoma [25, 26]. Here, we found that GALNT2 was upregulated in cervical cancer not only by using bioinformatic analysis, but also by using immunohistochemistry in different pathological types of cervical tissue. The protein level of GALNT2 was higher in SCC and HSIL than that in normal cervix and LSIL. It is worth noting that, there was no significant difference in IHC scores between HSIL and SCC. These results suggest that the carcinogenic effect of GALNT2 might play a role in the late stage of cervical lesions.

Previous researches have demonstrated that alterations in glycosyltransferases are correlated with survival outcome of cancer patients. For instance, low expressions of glycosyltransferase genes B4GALT1, EXT1, MGAT5B and POFUT1 predicted poor patient’s survival in bladder cancer [27]. The glycosyltransferase ST6Gal-I expression contributed to the poor survival in gastric cancer [9]. Glioblastoma multiforme patients with high expression of beta-1,3-N-acetylglucosaminyltransferase B3GNT5 had a worse overall survival [28]. High expression of beta-1,3-galactosyltransferase C1GALT1 had been identified as an independent prognostic factor for worse overall survival in gastric cancer [29]. In current study, we demonstrated that GALNT2 and several other GalNAc-T subfamily members (GALNT3, GALNT4, GALNT10, GALNT13 and GALNT15) were associated with worse survival rate of cervical cancer patients, and GALNT2 also predicted unfavorable prognosis in HNSC, KIRC, LUAD, LUSC, STAD, READ and UCEC. This observation indicates that GALNT2 may be a promising prognostic biomarker for patients with cervical cancer.

As a polypeptide GalNAc-transferase, GALNT2 mediates the O-glycosylation of the ANGPTL3 [30], influences the O-glycosylation and phosphorylation of EGFR [21], and regulates PI3K/Akt/mTOR axis and ectonucleotide pyrophosphatase phosphodiesterase 1 (ENPP1) expression [31, 32]. In lung adenocarcinoma, GALNT2 was found to regulate the proliferation and mobility of cancer cells via activating the Notch/Hes1-PTEN-PI3K/Akt axis [33]. Here, we showed that GALNT2 was not only significantly correlated with various carcinogenesis pathways, including KRAS signaling, epithelial mesenchymal transition, IL6/JAK/STAT3 signaling, inflammatory response, Notch signaling pathway and cytosolic DNA sensing pathway, but also associated with immune-related gene sets, such as cytokine binding, cytokine receptor binding, cytokine receptor activity, antigen binding, adaptive immune response and regulation of immune effector process. Previous studies have revealed that the expression of chemokines, immune checkpoints could be used as immunotherapeutic targets and prognostic biomarkers, and might inform treatment decision-making for cervical cancer [34–36]. Our data demonstrate that concomitant high expression of GALNT2 and cytokines (IL1A, IL1B, IL11), chemokines (CXCL1, CXCL2, CXCL5, CXCL6, CXCR1 or CCR3) or immune modulatory molecules (CD47, CD274, CD276, CSF1R, TNFSF9 and TNFSF11) could predict worse survival of cervical cancer patients, compared with their corresponding low or mixed expression groups. These results suggest that GALNT2 might help to predict the anti-tumor immune response of cervical cancer patients. However, additional functional experiments are needed to reveal more detailed information about the role of GALNT2 in anti-tumor immune response.

## Conclusion

In conclusion, our study revealed that the mRNA expression and the protein level of GALNT2 were increased in cervical cancer, and concomitant high expressions of GALNT2 and specific cytokines, chemokines or immune modulatory molecules could predicte patients’ unfavorable survival in cervical cancer. Collectively, our findings indicate that GALNT2 might be used as a potential prognostic biomarker for cervical cancer.

## Data Availability

The original contributions presented in the study are included in the article/Supplementary Material, further inquiries can be directed to the corresponding authors.

## Supplementary Material

Supplementary Figure S1
**(A) **The expression of GALNT2 in TCGA-CESC based on individual cancer stage in the UALCAN web site. **(B)** The expression of GALNT2 in TCGA-CESC based on tumor histology in the UALCAN web site. **(C)** The expression of GLANT2 in chemotherapy complete response, partial response, progressive disease and stable disease groups based on TCGA-CESC data. **(D)** The differential expression between tumor and adjacent normal tissues for GALNT2 across different types of cancers *via* TIMER platform. **p* < 0.05, ***p* < 0.01, ****p* < 0.001.

Supplementary Figure S2
**(A)** The biological pathways and processes potentially regulated by GALNT2 *via* Hallmark gene sets deposited in the GSEA. The normalized enrichment scores (NES) were shown. **(B)** GSEA enrichment plots showed that a series of Hallmark gene sets were enriched in the high or low expression of GALNT2 group.

Supplementary Figure S3 Differences in the infiltration levels of 28 immune cell types between high- and low-GALNT2 group in TCGA cohort and GSE44001 cohort. **p* < 0.05; ***p* < 0.01; ****p* < 0.001.

Supplementary Figure S4 Kaplan-Meier curves were plotted based on the different groups of GALNT2/IL1B **(A)**, GALNT2/IL11 **(B)**, GALNT2/CXCL5 **(C)** and GALNT2/CXCL6 **(D)** in TCGA cohort and GSE44001 cohort.

Supplementary Figure S5
**(A)** Kaplan-Meier curves of OS for patients with cervical cancer stratified by the expression of different immune modulatory molecules (CD47, CD274, CD276, CSF1R, TNFSF9, and TNFSF11).

Supplementary Figure S6 Kaplan-Meier curves were plotted based on the different groups of GALNT2/CD276 **(A)** and GALNT2/CSF1R **(B)** in TCGA cohort and GSE44001 cohort.
