# Supplementary figures and images for: O-Glycosylating Enzyme GALNT2 Predicts Worse Prognosis in Cervical Cancer
Source: Pathol Oncol Res. 2022 Aug 30;28:1610554. doi: 10.3389/pore.2022.1610554 (PMC9469784; doi:10.3389/pore.2022.1610554)

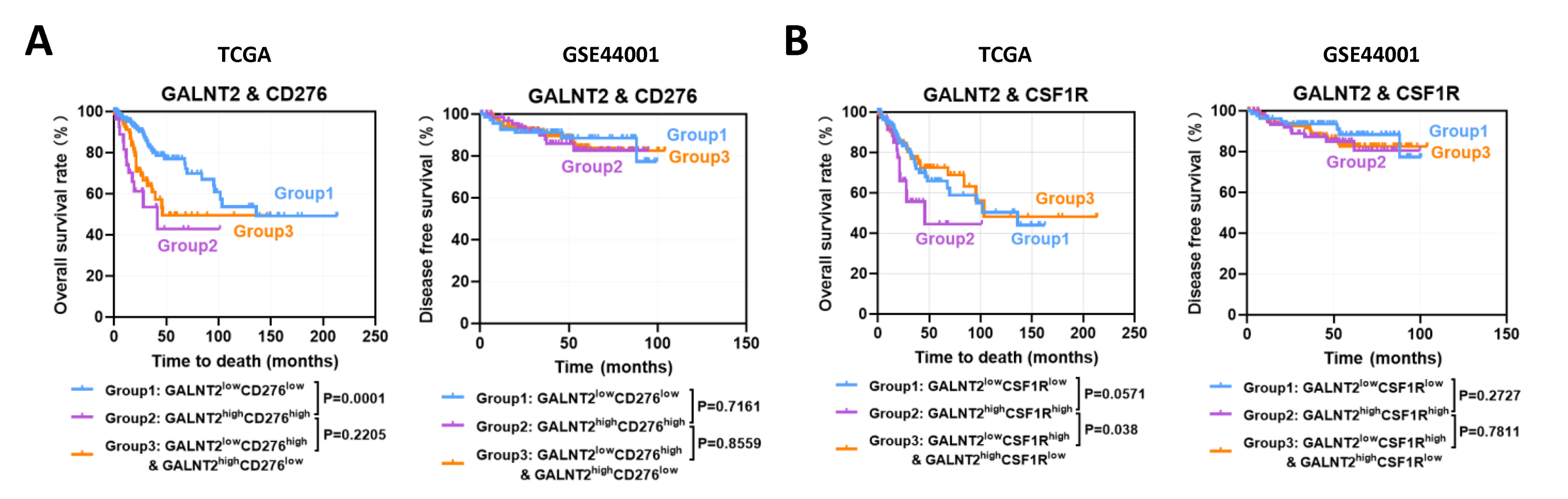

Supplement: Supplementary file 1 [file Image6.TIF]

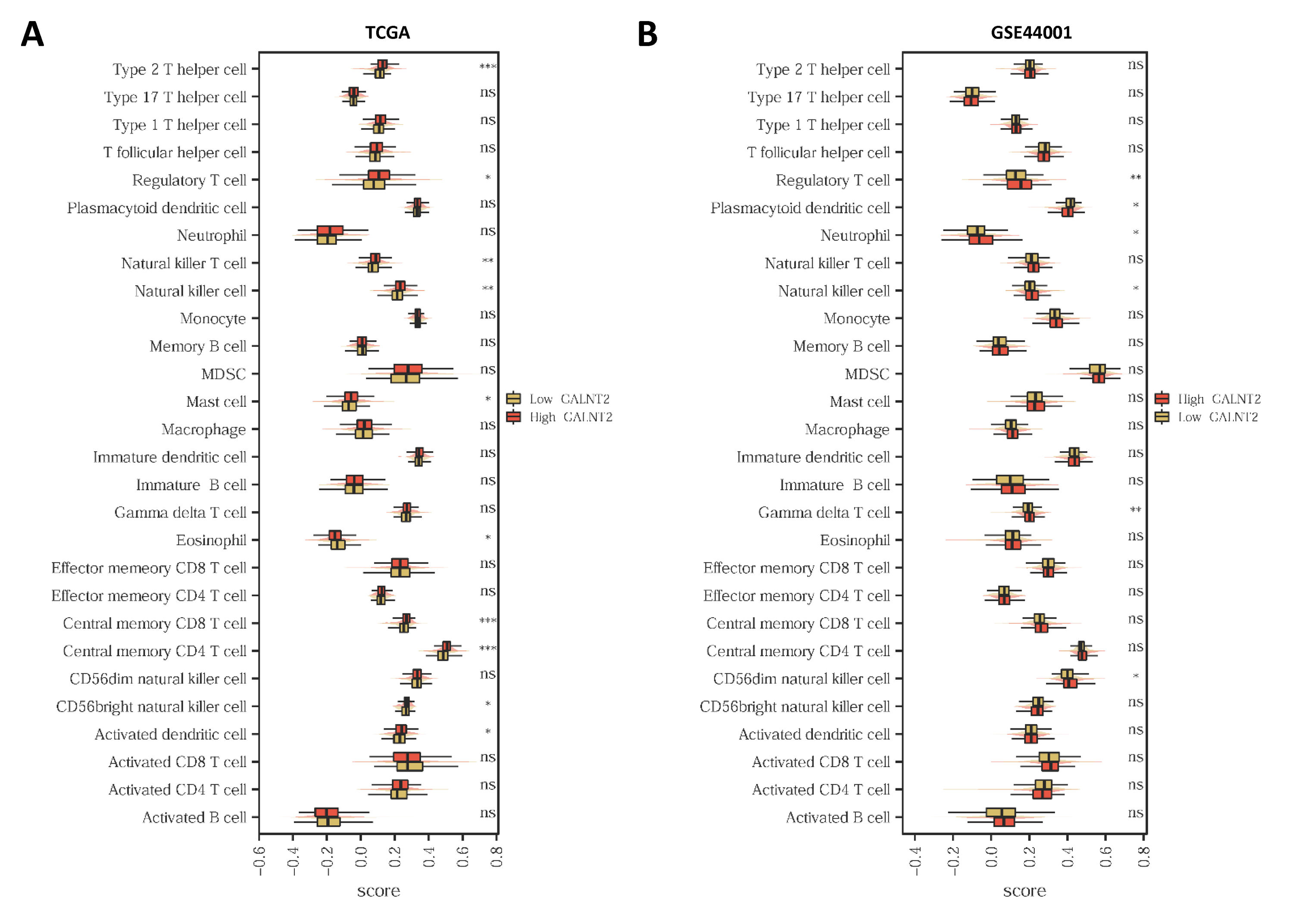

Supplement: Supplementary file 3 [file Image3.TIF]

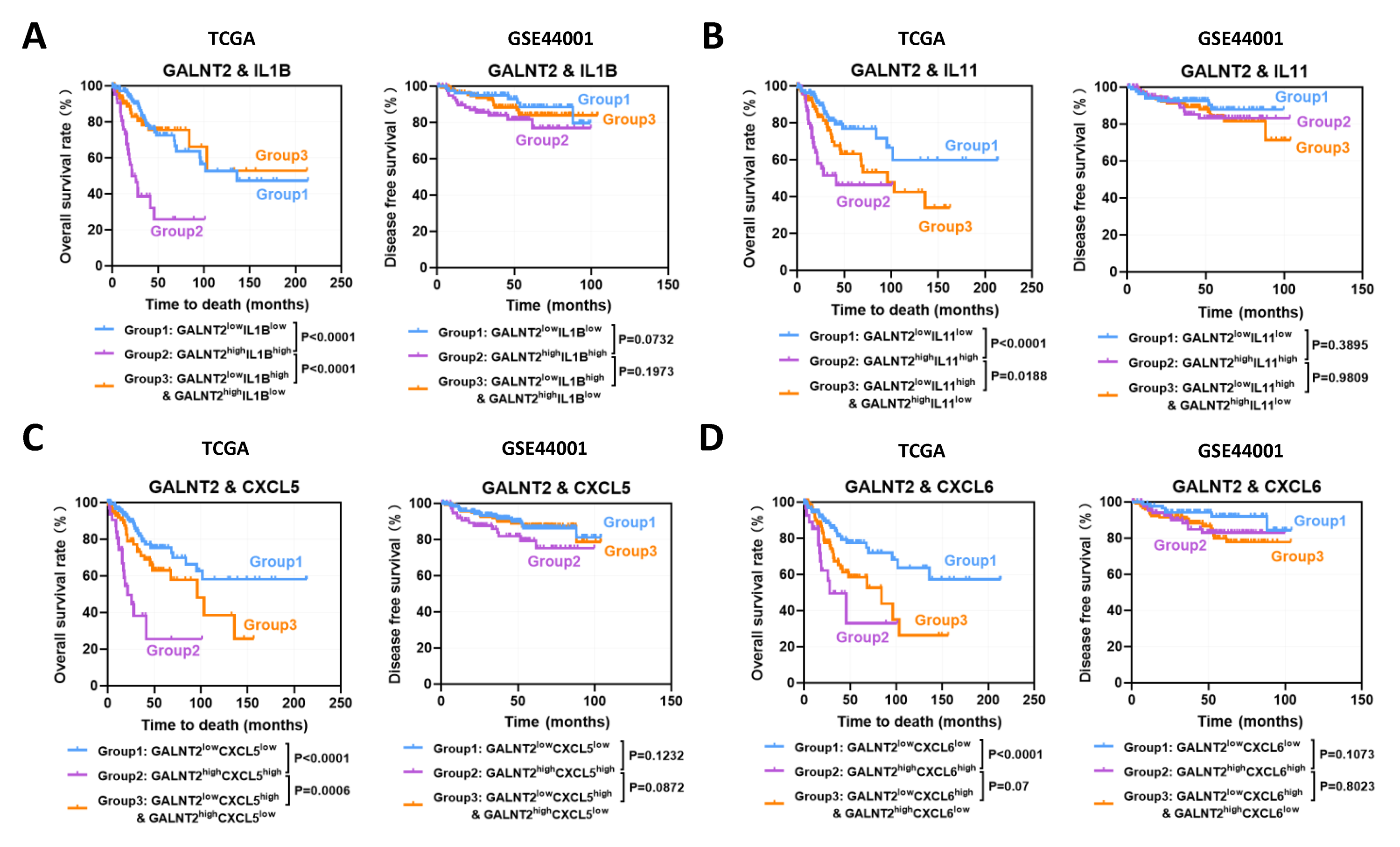

Supplement: Supplementary file 4 [file Image4.TIF]

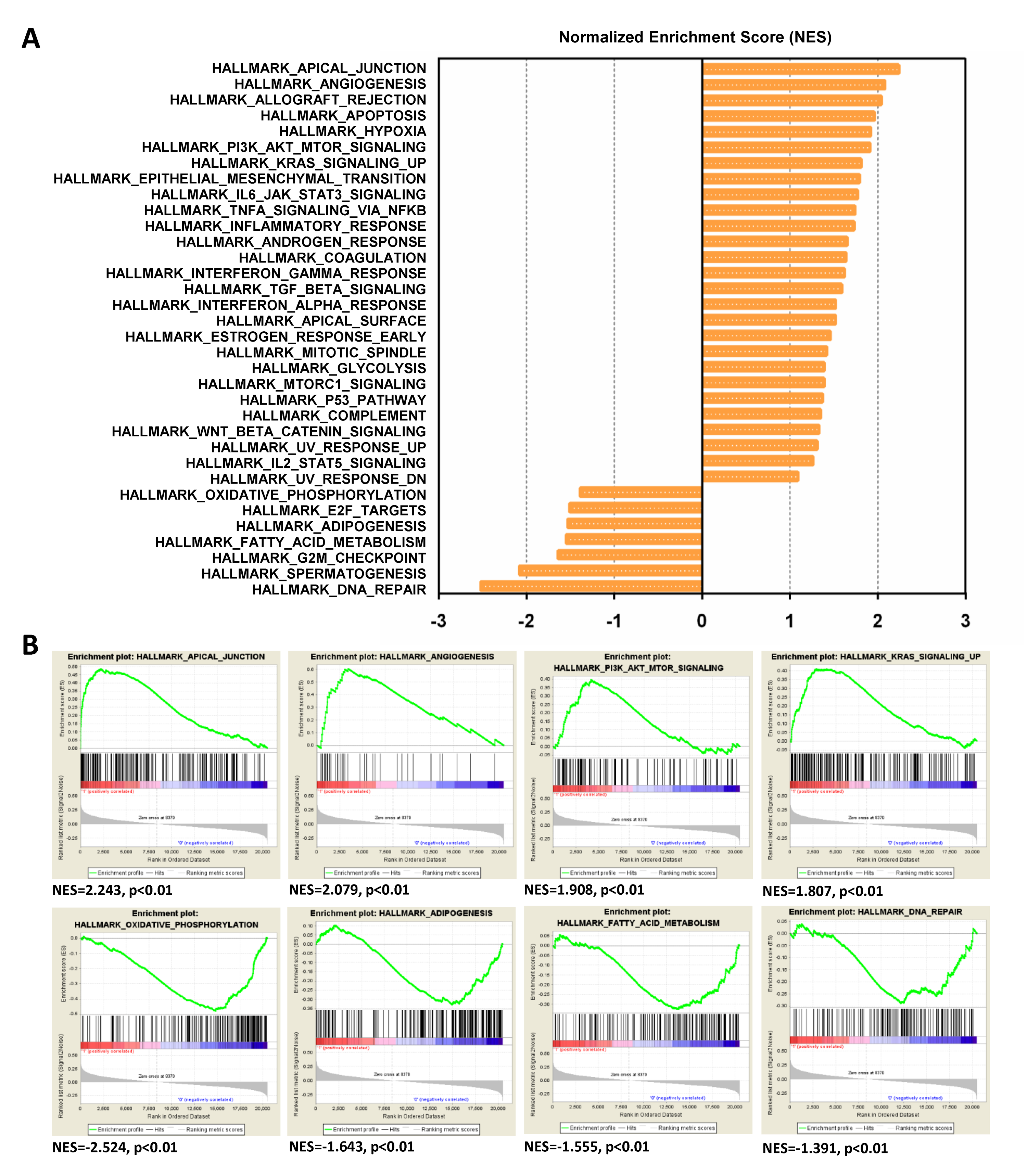

Supplement: Supplementary file 5 [file Image2.TIF]

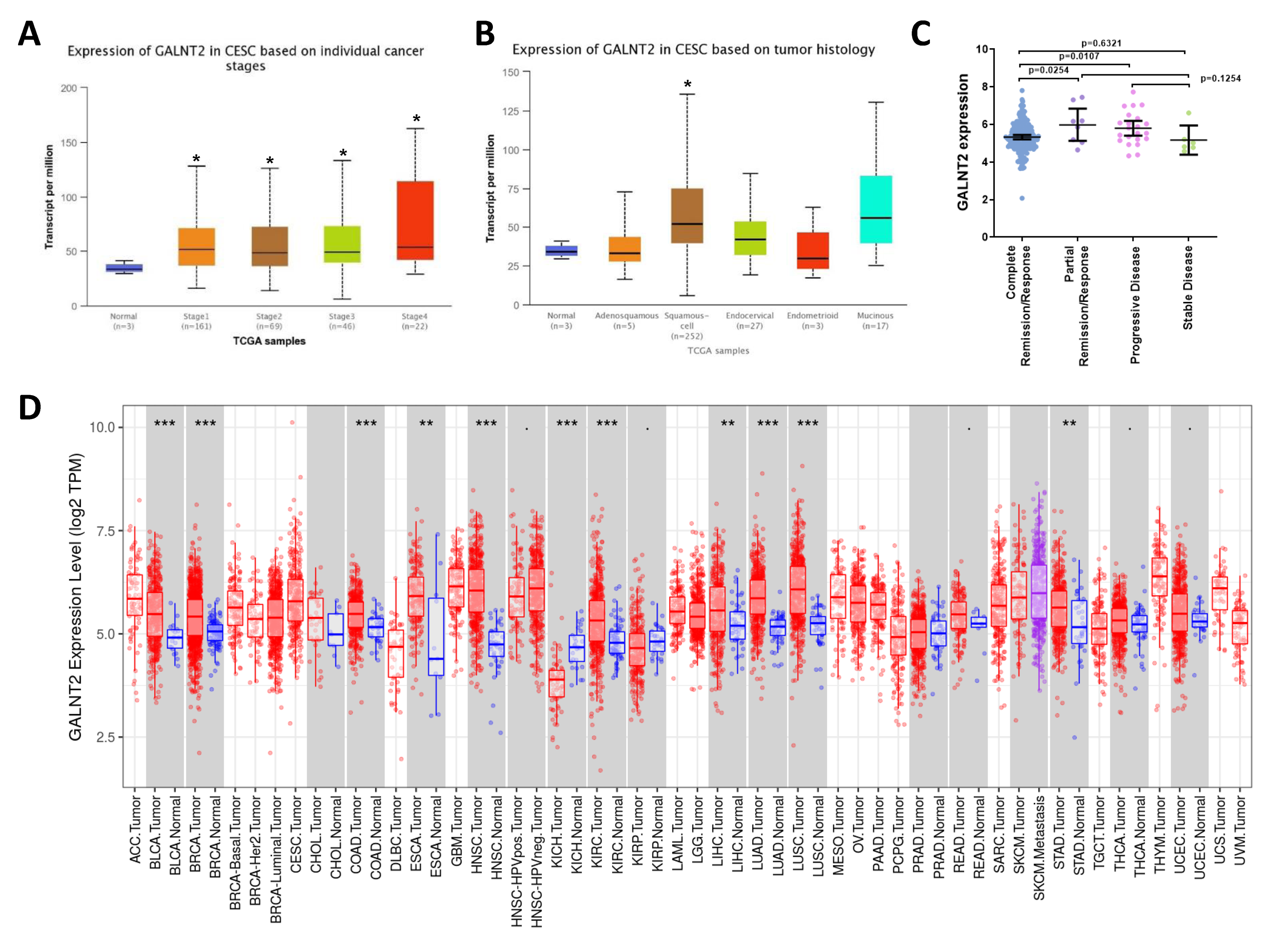

Supplement: Supplementary file 6 [file Image1.TIF]

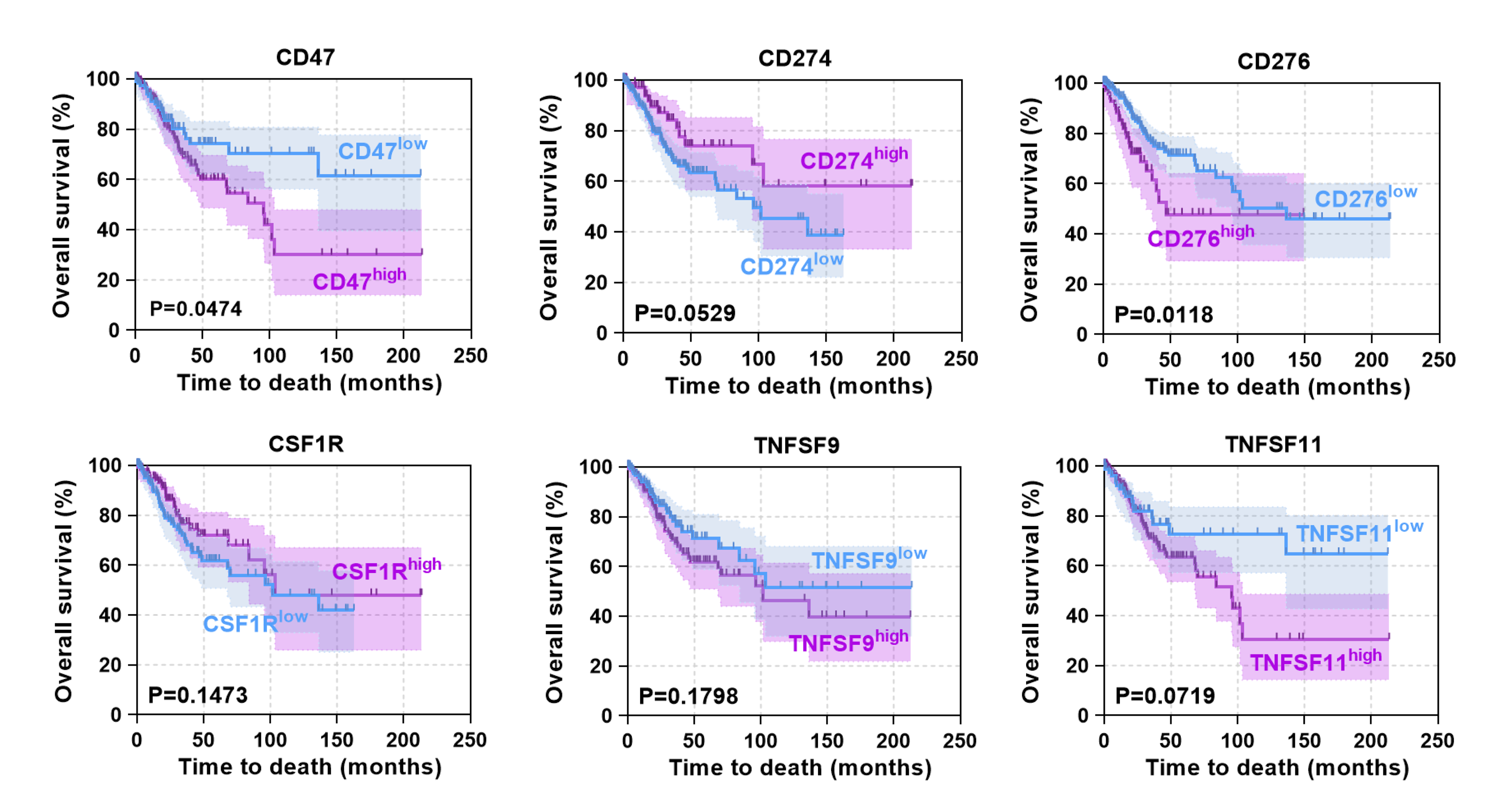

Supplement: Supplementary file 8 [file Image5.TIF]
