# Supplementary material for: O-Glycosylating Enzyme GALNT2 Predicts Worse Prognosis in Cervical Cancer
Source: Pathol Oncol Res. 2022 Aug 30;28:1610554. doi: 10.3389/pore.2022.1610554 (PMC9469784; doi:10.3389/pore.2022.1610554)
Supplement: Supplementary file 2 [file Table1.DOCX]

Supplementary Table S1. The known biological roles of immune-modulatory molecules used in current study.

| Molecules | Biological roles |
| --- | --- |
| CCR3 | CCR3 is a receptor for C-C type chemokines. It belongs to family 1 of the G protein-coupled receptors. CCR3 binds and responds to a variety of chemokines, including CCL11, CCL26, CCL7, CCL13 and CCL5. |
| CXCL1 | CXCL1 is a secreted growth factor that signals through the G-protein coupled receptor, CXC receptor 2. CXCL1 plays a role in inflammation and as a chemoattractant for neutrophils. |
| CXCL2 | CXCL2 is part of a chemokine superfamily that encodes secreted proteins involved in immunoregulatory and inflammatory processes. |
| CXCL5 | CXCL5 is a member of the CXC subfamily of chemokines. CXCL5 is proposed to bind the G-protein coupled receptor chemokine receptor 2 to recruit neutrophils, to promote angiogenesis and to remodel connective tissues. |
| CXCL6 | CXCL6 is a member CXC chemokine family, and controls the functional topography of interleukin-22 producing intestinal innate lymphoid cells |
| CXCR1 | CXCR1 is a receptor for interleukin 8 (IL8), binds to IL8 with high affinity, and transduces the signal through a G-protein activated second messenger system. |
| IL11 | IL11 is shown to stimulate the T-cell-dependent development of immunoglobulin-producing B cells. |
| IL1A | IL1A is a pleiotropic cytokine involved in various immune responses, inflammatory processes, and hematopoiesis. |
| IL1B | IL1B is an important mediator of the inflammatory response, and is involved in a variety of cellular activities, including cell proliferation, differentiation, and apoptosis. |
| CD274 | CD274, also commonly referred to as PDL1, is a ligand that binds with the receptor PD1, and acts to block T-cell activation. |
| CD276 | CD276 belongs to the immunoglobulin superfamily, and thought to participate in the regulation of T-cell-mediated immune response. |
| CD47 | CD47 is a receptor for the C-terminal cell binding domain of thrombospondin, and it plays a role in membrane transport and signal transduction. |
| CSF1R | CSF1R is the receptor for colony stimulating factor 1, a cytokine which controls the production, differentiation, and function of macrophages. |
| TNFSF9 | TNFSF9 and its receptor are involved in the antigen presentation process and in the generation of cytotoxic T cells. |
| TNFSF11 | TNFSF11 was shown to be a dendritic cell survival factor and is involved in the regulation of T cell-dependent immune response. |
