# Supplementary material for: O-Glycosylating Enzyme GALNT2 Predicts Worse Prognosis in Cervical Cancer
Source: Pathol Oncol Res. 2022 Aug 30;28:1610554. doi: 10.3389/pore.2022.1610554 (PMC9469784; doi:10.3389/pore.2022.1610554)
Supplement: Supplementary file 7 [file Table2.DOCX]

Supplementary Table S2. Univariate and multivariate Cox regression analyses.

|  | Univariate Cox | | | Multivariate Cox | | |
| --- | --- | --- | --- | --- | --- | --- |
|  | Hazard Ratio | 95%CI | P-value | Hazard Ratio | 95%CI | P-value |
| GALNT2 | 1.66 | 1.31-2.1 | 3.00e-05 | 1.48 | 1.07-2.03 | 0.01647 |
| CCR3 | 1.07 | 0.97-1.19 | 0.17084 | NA | NA | NA |
| CD274 | 0.93 | 0.81-1.07 | 0.33398 | NA | NA | NA |
| CD276 | 1.21 | 0.91-1.6 | 0.19505 | NA | NA | NA |
| CD47 | 1.05 | 0.75-1.47 | 0.77699 | NA | NA | NA |
| CSF1R | 0.95 | 0.8-1.14 | 0.57753 | NA | NA | NA |
| TNFSF11 | 1.1 | 0.99-1.22 | 0.0895 | NA | NA | NA |
| TNFSF9 | 1 | 0.88-1.14 | 0.99618 | NA | NA | NA |
| CXCL1 | 1.12 | 1-1.25 | 0.04727 | 0.97 | 0.83-1.14 | 0.70997 |
| CXCL2 | 1.17 | 1.04-1.32 | 0.00681 | 1.16 | 0.97-1.39 | 0.09435 |
| CXCL5 | 1.11 | 1.03-1.19 | 0.00518 | 1.04 | 0.94-1.14 | 0.46528 |
| CXCL6 | 1.04 | 0.96-1.13 | 0.31092 | NA | NA | NA |
| CXCR1 | 1.13 | 1.01-1.26 | 0.02852 | 0.99 | 0.87-1.14 | 0.94121 |
| IL11 | 1.18 | 1.03-1.35 | 0.01491 | 1 | 0.85-1.17 | 0.98979 |
| IL1A | 1.07 | 0.98-1.17 | 0.13221 | NA | NA | NA |
| IL1B | 1.22 | 1.07-1.39 | 0.00281 | 1.01 | 0.85-1.2 | 0.92452 |
| stage | 2.27 | 1.33-3.88 | 0.0027 | 2.64 | 1.46-4.77 | 0.00131 |
| grade | 0.92 | 0.54-1.55 | 0.74998 | NA | NA | NA |
| age | 1.02 | 1-1.04 | 0.11743 | NA | NA | NA |
